# Supplementary material for: Determinants of Smoking Cessation Outcomes and Reasons for Relapse in Patients Admitted to a Smoking Cessation Outpatient Clinic in Turkey
Source: Int J Environ Res Public Health. 2024 Mar 7;21(3):310. doi: 10.3390/ijerph21030310 (PMC10970556; doi:10.3390/ijerph21030310)
Supplement: Supplementary file 1 [file ijerph-21-00310-s001.zip › ijerph-2823670-supplementary.pdf]

Supplementarty Material

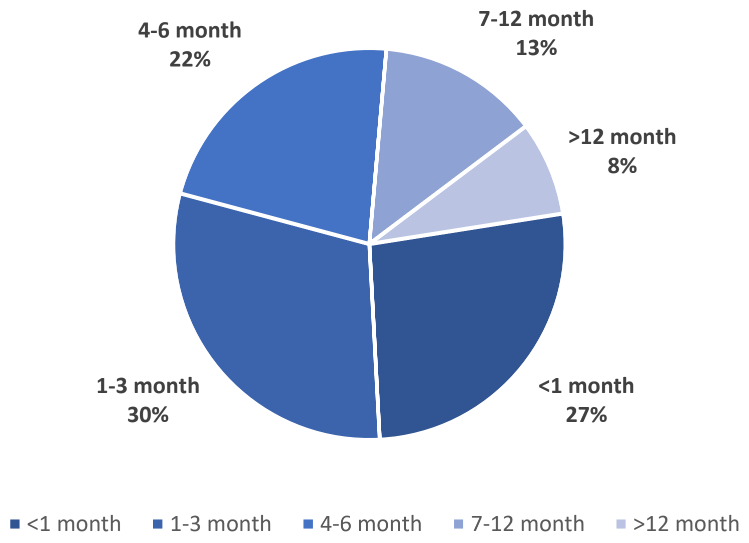

Figure S1. The duration of time after treatment before smoking reuptake in the smoking cessation group.

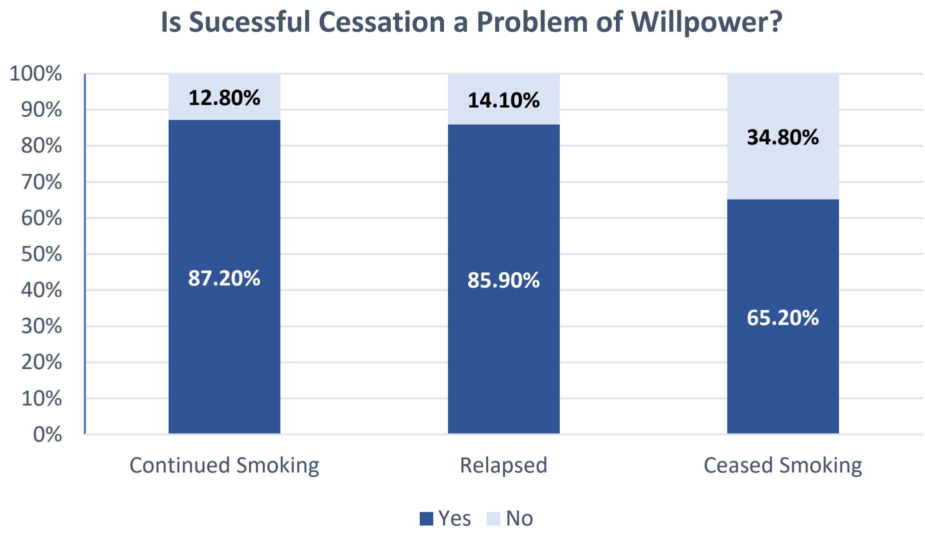

Figure S2. Differeces in the proportion of patients who percieve successful smoking cessation as a problem of willpower across groups.

Supplementary Table S1. Demographic characteristics of participants who successfully ceased smoking compared to participants who were unsuccessful (continued smoking or relapsed)

1. Why do people smoke? Is smoking a disease? What kind of disease is it?
2. What do you think about getting professional help to quit smoking?
3. What are the reasons for of restarting the smoking and relapse of the disease?
4. Let's go back to the moment when you smoked your first cigarette after quitting, please close your eyes and live that moment. What do you remember, how do you feel?
5. When you smoked that first cigarette, did you think you would continue to smoke?
6. What if you wouldn't have smoked "that first cigarette"?
7. Were you informed about relapses while receiving treatment? Do you think that being informed works or will help?
8. What obstacles you faced in continuing not to smoke?
9. How often, in which situations and environments did you want to smoke? How did you cope? (or couldn't cope?)
10. Do you want to quit smoking again? Do you have any plans for this? (1 month, 6 months…)

Supplementary Table S2. Demographic characteristics of participants who successfully ceased smoking compared to participants who were unsuccessful (continued smoking or relapsed)

	**Total**	**Succesful Cessation**	**Unsuccessful Cessation**	**p-value**
Age				0,957†
18-25 years	6 (%3,4)	2 (%4,3)	4 (%3,0)	
26-40 years	70 (%39,1)	17 (%37,0)	53 (%39,9)	
41-65 years	96 (%53,6)	26 (%56,5)	70 (%52,6)	
>65 years	7 (%3,9)	1 (%2,2)	6 (%4,5)	
Gender				0,606‡
Male	105 (%58,7)	25 (%54,3)	80 (%60,2)	
Female	74 (%41,3)	21 (%45,7)	53 (%39,8)	
Marital status				0,449‡
Single	52 (%29,4)	11 (%23,9)	41 (%31,3)	
Married	125 (%70,6)	35 (%76,1)	90 (%68,7)	
Education status				0,335†
Elementary and below	19 (%10,7)	4 (%8,7)	15 (%11,4)	
Secondary education	73 (%41,0)	17 (%37,0)	56 (%42,4)	
University	86 (%48,3)	25 (%54,3)	61 (%46,2)	
Concomitant disease	*n=176*	*n=46*	*n=130*	
No	78 (%44,3)	16 (%34,8)	62 (%47,7)	0,180‡
CSD	30 (%17,0)	10 (%21,7)	20 (%15,4)	0,449‡
Respiratory	24 (%13,6)	11 (%23,9)	13 (%10,0)	**0,035‡**
Psychiatric	16 (%9,1)	4 (%8,7)	12 (%9,2)	>0,999 ¶
malignancy	2 (%1,1)	1 (%2,2)	1 (%0,8)	0,456¶
Other	55 (%31,3)	14 (%30,4)	41 (%31,5)	>0,999 ‡
Alcohol history				0,512‡
No	109 (%62,3)	31 (%67,4)	78 (%60,5)	
Yes	56 (%37,7)	15 (%32,6)	51 (%39,5)	

† Mann Whitney U test, ‡ Chi-Square test with continuity correction, ¶ Fisher's exact test. Bold values indicates significant results.

Table S3. Reported reasons for smoking reuptake in patients who relapsed.

Reason for Reuptake	**n=93**
Stress	35 (%37,6)
Being in a smoking environment	26 (%28,0)
Sadness	21 (%22,6)
Severe withdrawal	16 (%17,2)
Anger	7 (%7,5)
Automated behavior	5 (%5,4)
Other	2 (%2,2)

Table S4. Differences in their desire to quit, perceived obstacles and time until next quit attempt between those who continued smoking and those relapsed.

	**Total**	**Continued Smoking**	**Relapsed to Smoking**	**p-value**
Desire to quit				**0,006†**
Wants to quit	113 (%86,3)	29 (%72,5)	84 (%92,3)	
Doesn't want to quit	18 (%13,7)	11 (%27,5)	7 (%7,7)	
Obstacles to quitting				
None	16 (%12,4)	2 (%5,1)	14 (%15,6)	0,146‡
Sadness	6 (%4,7)	4 (%10,3)	2 (%2,2)	0,068‡
Stress	55 (%42,6)	16 (%41,0)	39 (%43,3)	0,960†
Will	16 (%12,4)	5 (%12,8)	11 (%12,2)	>0,999‡
Habit	23 (%17,8)	8 (%20,5)	15 (%16,7)	0,784†
Thinking that treatment is useless	9 (%7,0)	6 (%15,4)	3 (%3,3)	**0,022‡**
Not feeling ready	4 (%3,1)	1 (%2,6)	3 (%3,3)	>0,999‡
See as a friend	4 (%3,1)	0 (%0,0)	4 (%4,4)	0,314‡
Enjoyment	7 (%5,4)	3 (%7,7)	4 (%4,4)	0,431‡
Other	6 (%4,7)	0 (%0,0)	6 (%6,7)	0,177‡
When are they thinking about quitting?				0,139¶
Now	44 (%38,3)	18 (%60,0)	26 (%30,6)	
<1 month	27 (%23,5)	2 (%6,7)	25 (%29,4)	
1-3 months	18 (%15,7)	1 (%3,3)	17 (%20,0)	
4-12 months	10 (%8,7)	4 (%13,3)	6 (%7,1)	
>12 months	16 (%13,9)	5 (%16,7)	11 (%12,9)	

† Continuity-corrective Chi-Square test, ‡ Fisher's probability test with exact results, ¶ Mann Whitney U test. Bold values indicates significant results.

Table S5. Within and between group differences in the difficulty levels of smoking cessation before and after treatment

	Before	After	p-value †	Change
Relapsed to Smoking	10 (5-10)	3,5 (0-10)	**<0,001**	6.-5 (-10 – 1)
Ceased Smoking	9,5 (3-10)	3 (0-10)	**<0,001**	-5.5 (-10 – 1)
p-value ‡	0,427	0,496	-	0,928

Data; median (minimum - maximum), † Comparisons between pre-treatment and post-treatment within the groups, Wilcoxon Sign test, results for p<0.025 according to Bonferroni Correction were considered statistically significant, ‡ Comparisons between the group that quit smoking after treatment and the group that quit smoking and restarted, Mann Whitney U test, Bonferroni in comparisons between groups before and after treatment According to the correction< the results for p<0.025 were considered statistically significant, while the results for p0.05 were considered statistically significant in the comparison made in terms of change scores. Bold values indicates significant results.

Table S6. Distribution of factors predicting successful cessation in ceased smoking group across the relapsed to smoking group.

	Total (n=133)	Relapsed to Smoking (n=88)	Ceased Smoking (n=45)	p-value
Medication support	61 (%45,9)	41 (%46,6)	20 (%44,4)	0,959†
Individual motivation	79 (%59,4)	49 (%55,7)	30 (%66,7)	0,301†
Health anxiety	26 (%19,5)	11 (%12,5)	15 (%33,3)	**0,008†**
Social motivation	16 (%12,0)	8 (%9,1)	8 (%17,8)	0,240†
Other	4 (%3,0)	2 (%2,3)	2 (%4,4)	0,604‡

† Chi-Square test with continuity correction, ‡ Fisher's probability test with definite results. Bold values indicates significant results.

Table S7. Distribution of side effects in the sample and across participants that continued smoking, relapsed to smoking and ceased smoking following treatment

	Total (n=167)	Continued Smoking (n=32)	Relapsed to Smoking (n=89)	Ceased Smoking (n=46)	p-value
Side effect	70 (%41,9)	15 (%46,9)	35 (%39,3)	20 (%43,5)	0,735†
Nausea	23 (%13,8)	5 (%15,6)	13 (%14,6)	5 (%10,9)	0,790†
Sleep disturbance	9 (%5,4)	3 (%9,4)	3 (%3,4)	3 (%6,5)	0,417‡
Gastrointestinal	12 (%7,2)	2 (%6,3)	7 (%7,9)	3 (%6,5)	0,935‡
Abnormal dreaming	6 (%3,6)	1 (%3,1)	3 (%3,4)	2 (%4,3)	0,949‡
Headache	4 (%2,4)	0 (%0,0)	1 (%1,1)	3 (%6,5)	0,100‡
Other	36 (%21,6)	9 (%28,1)	17 (%19,1)	10 (%21,7)	0,567†

† Pearson's Chi-Square test, ‡ Likelihood Ratio test.

Table S8. Negative and positive changes experienced by ceased smoking and relapsed to smoking groups following treatment

	**Total**	**Relapsed to Smoking**	**Ceased Smoking**	**p-value**
Negative change	*n=133*	*n=88*	*n=45*	
None	54 (%40,6)	36 (%40,9)	18 (%40,0)	>0,999†
Weight gain	32 (%24,1)	16 (%18,2)	16 (%35,6)	**0,045**†
Intraoral wound	14 (%10,5)	9 (%10,2)	5 (%11,1)	>0,999‡
Irritability	29 (%21,8)	22 (%25,0)	7 (%15,6)	0,305†
Sorrow	21 (%15,8)	12 (%13,6)	9 (%20,0)	0,483†
Other	5 (%3,8)	4 (%4,5)	1 (%2,2)	0,662‡
Positive change	*n=130*	*n=85*	*n=45*	
None	13 (%10,0)	10 (%11,8)	3 (%6,7)	0,541‡
Physical performance	80 (%61,5)	48 (%56,5)	32 (%71,1)	0,149†
Taste, smell	44 (%33,8)	27 (%31,8)	17 (%37,8)	0,621†
Sleep quality	18 (%13,8)	12 (%14,1)	6 (%13,3)	>0,999†
Cosmetic causes	26 (%20,0)	13 (%15,3)	13 (%28,9)	0,107†
Other	31 (%23,8)	18 (%21,2)	13 (%28,9)	0,444†

† Chi-Square test with continuity correction, ‡ Fisher's exact test. Bold values indicates significant results.
